# Effect of the pigmentation, shine, weight, and shape index of the quail egg (*Coturnix coturnix japonica*) on the hatchability rate

**DOI:** 10.5455/javar.2021.h554

**Published:** 2021-11-02

**Authors:** Ernestina Gutiérrez, Gerardo Ordaz, Rosa Elena Pérez, Ruy Ortiz, Aureliano Juárez

**Affiliations:** 1 Instituto de Investigaciones Agropecuarias y Forestales-UMSNH, Tarímbaro, México; 2 Centro Nacional de Investigación Disciplinaria en Fisiología y Mejoramiento Animal-INIFAP, Ajuchitlán, México; 3 Facultad de Químico Farmacobiología-UMSNH, Morelia, México; 4 Facultad de Medicina Veterinaria y Zootecnia-UMSNH, Morelia, México

**Keywords:** *Poultry*, *hatching*, *incubation*, *cotorniculture*

## Abstract

**Objective:**

The influence of the degree of pigmentation, shine, weight, and form index on the hatchability rate of quail eggs (*Coturnix coturnix japonica*) was examined.

**Materials and Methods:**

Three thousand three hundred eggs were incubated after they were classified according to the degree of shell pigmentation: high (HP), medium (MP), and low (LP); shell shine: shiny (SS), and opaque; Egg weight: <11, 11.0–11.9, 12.0–12.9, and >13 gm; form index: <78% and >78%. The Mann–Whitney non-parametric test for independent samples was used to evaluate hatching data.

**Results:**

Hatchability was best in eggs with HP and SS (*p* < 0.05): 69.2% and 75.7%, respectively; eggs with MP had the lowest hatchability rate (63.1%). The highest risk of embryo death (17.8%) was observed in eggs containing LP (*p* < 0.05). Eggs weighing between 12 and 12.9 gm had the highest hatchability rate (p < 0.05) (79.11%). Eggs with a form index >78% had the highest hatchability rate (*p* < 0.05) (67.62%).

**Conclusions:**

HP, MP shell eggs, and SS eggs with a weight of between 12.0 and 12.9 gm and a shape index of greater than 78% have a higher hatchability rate.

## Introduction

The quail (*Coturnix coturnix japonica*), a native of East Asia, is now found worldwide. The quick growth, rusticity, and precocity of this species have made it a popular species for introduction into meat or quail egg production systems [1], features that define quail production as a versatile industry adaptable to a range of production systems. It is worth noting that regardless of the system type (intensive, semi-intensive, or family), the primary goal of production is the sale of meat and eggs [2]. The egg production is more critical, as the quail egg is not only utilized for human food, but also as the raw material for the development of quail chicks for the system or for export [3].

In terms of quail egg production, these are white and coated in dots ranging from brown to black, passing through blue or green [4]. The color variation in egg spots is determined by the amounts of three fundamental organic components: protoporphyrin, biliverdin, and zinc chelate [5]. For example, biliverdin and biliverdin chelate with zinc have a higher proportion in eggs with blue or green spots, but protoporphyrin predominates in eggs with brown spots [6]. Biliverdin and protoporphyrin are both produced in the oviduct gland and then deposited in the eggshell concurrently [7]. The color of the eggshell has been reported to affect both its quality and biological value [8–10]. This is even though environmental influences such as relative humidity and storage time cannot be ruled out [11].

Apart from the shell color, the morphological characteristics of the quail egg also influence the hatchability and growth of the embryo [10]. According to several findings, hatching eggs with a smooth, shiny surface have a higher hatchability rate [9]. This attribute is related to the cuticle’s quality, which protects them from dehydration and infection. In comparison, Yilmaz et al. [12] state that non-shiny eggs are unsuitable for hatching due to their increased porosity, endangering the embryo’s viability. As a result, non-shiny eggs are related to decreased mineralization and increased contamination rates, both of which impair embryo survival [13].

In terms of the morphology and hatchability of the quail egg (shape, weight, pigmentation, and shell thickness), it has been demonstrated that eggs with thicker shells have a greater hatchability rate (+9.0%) [14–16]. There are reports of a link between the level of shell pigmentation and the thickness of the shell and its hatchability, implying a probable correlation between shell pigmentation processes and calcification [15]. However, there is a dearth of studies examining the fertility of this species in relation to the egg’s morphological traits [17]. Thus, the purpose of this inquiry was to determine the effect of shell coloration and shine, egg weight (EW), and form index on the hatchability rate of quail eggs.

## Materials and Methods

### Ethical approval

The Technical Scientific Committee of the Faculty of Veterinary Medicine and Zootechnics (FVMZ) of the Universidad Michoacana de San Nicolás de Hidalgo evaluated and approved the protocol (UMSNH). Animal handling followed the Official Mexican Standard for the Production, Care, and Use of Laboratory Animals [18] as well as the International Guiding Principles for Biomedical Research Involving Animals [19].

### Experimental site

The research was carried out at the FMVZ-UMSNH poultry sector, which is located at 9.5 km of the Morelia Zinapécuaro road in the Tarimbaro municipality of Michoacan, Mexico, between the coordinates 19°47’11” of North latitude and 101°10’35” of West longitude, at an elevation of this sector is classed as a semi-intensive quail egg producing system based on its infrastructure and equipment characteristics. 

### Experimental design and research development

To accomplish the research objective, viable eggs (*n* = 3,300) were collected from a commercial quail (*C. coturnix japonica*) production system in Morelia, Michoacan, Mexico. Every 2 weeks, eggs were retrieved, and during a 6-week period, 1,100 fertile eggs were gathered via visit. This system’s breeding population is comprised of 10,000 36-week-old birds. The quails were housed in a battery cage system in a one (♂) to three (♀) sex ratio. The birds were provided with water and feed *ad libitum*, and the diet contained 2,900 kcal of ME/kg and 20% crude protein.

The eggs were packaged in special cones for shipment to the FMVZ-UMSNH poultry sector, where they were graded according to their degree of coloration (proportion of spots, shine, weight, and shape index of the egg). The eggs were identified and grouped according to their pigmentation level: high pigmentation (HP) was greater than 14 cm^2^, medium pigmentation (MP) was between 8 and 13.9 cm^2^, and low pigmentation (LP) was less than 8 cm^2^. The pigmented surface area (cm^2^) was estimated using the transparent millimeter paper method, which entails laying the paper on the surface to be measured and tracing the contour; 1, 1/2, and 1/4 cm squares are then tallied and summed to determine the pigmented area [20].

Regarding the shell shine, it was classified as follows: shiny (SS) or opaque (OS) shell. For egg classification according to weight (EW), it was classified into four categories (Ct): Ct 1, <11.0 gm; Ct 2, 11.1–11.9 gm; Ct 3, 12.0–12.9 gm; and Ct 4, >13 gm. For this, the eggs were weighed with a digital scale with a precision of 0.1 gm. The shape index (SI) was determined with the longitudinal and transverse measurements of the eggs through the following equation:


$$\mathrm{Shape}\;\mathrm{Index}\;(\mathrm{SI})=\frac{\mathrm{Cross}\;\mathrm{diameter}}{\mathrm{Longitudinal}\;\mathrm{diameter}}\times 100$$

According to the results obtained from the SI, they were classified into two groups: SI < 78% and SI > 78%.

The eggs were incubated in three electric incubators with a capacity of 300 eggs each; the incubation temperature was 37.5°C to 38°C and the humidity level was 60% to 65%. The picked egg was disinfected with a 3% iodine solution before incubation. Following that, the eggs were individually recognized and placed in mesh bags according to their categorization. The same operator rotated the eggs manually (three times a day). The eggs were delivered to hatcheries after 15 days of incubation.

### Statistical analysis

SAS 9.4® statistical software was used to evaluate and statistically process the experimental data. For independent samples, the *F* test and student’s *t* test were used to compare the groups. After confirming the distribution’s normality with the Kolmogorov-Smirnov tests [21], the hatching data was analyzed using the non-parametric test and Mann–Whitney [22] for independent samples. The computed regression equation was equal to zero to establish the critical point between EW and hatching rate. The least-squares means (LS means) approach was used to determine the differences across groups. The significance level for differences between groups was set at α < 0.05. The results are expressed as the mean standard error of the mean.

**Table 1.** LS means for the hatchability rate (%) according to the color and shine of the eggshell.

Hatchability rate	Eggshell color			Eggshell shine	
	HP	MP	LP	SS	OS
*N*	280	292	305	300	300
Hatched	69.2^ac^	63.1^a^	50.0^b^	75.7^c^	56.4^b^
Not hatched	7.7^a^	13.1^b^	17.8^c^	6.10^a^	10.2^b^
Dead embryo	10.2^a^	10.5^a^	17.8^b^	12.1^a^	10.2^a^
Fertile	12.8^a^	13.1^b^	14.3^b^	6.1^a^	20.5^b^
Contaminated	0.0^a^	0.0^a^	0.0^a^	0.0^a^	2.6^b^
SEM	3.3	3.3	3.2	5.9	5.9

HP = high pigmentation; MP = medium pigmentation; LP = low pigmentation; SS = shiny shell; OS = opaque shell.

^a,b,c^ Different literals indicate statistical difference (*p* < 0.05) within the row.

## Results

The effect of shell color and shine on markers of egg hatchability (Table 1) revealed that eggs with SS and HP had the highest hatching rate (*p* < 0.05) (Table 1). Despite the fact that there was no difference in hatching rate (*p* > 0.05) between SS (69.2%) and HP (75.7%). SS eggs hatched slightly faster than HP eggs: 6.5% more hatching (Table 1). The hatching rate of eggs containing OS and LP was significantly lower (*p* < 0.05) (Table 1).

According to the color and shine of the shell, the rate of dead embryos was significantly higher (p < 0.05) in eggs with LP (17.8%); the other color and shine classes of the shell showed no difference (p > 0.05): the rate of dead embryos was found to vary between 10.2% and 12.1% (Table 1). In terms of fertility rates, eggs with MP, LP, and OS had the lowest (p < 0.05) fertility percentages: 86.9%; 85.7%; and 79.5%, respectively; this was in comparison to eggs with SS and HP, which had an average fertility rate of 90.5% (Table 1).

**Figure 1.**
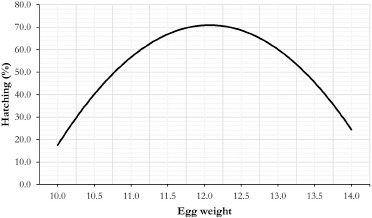
Prediction curve for hatching percentage according to egg weightEW. *Y*=−1,748.58+301.50*X*−12.49*X *^2^;R^2^=0.67;Critical point(12.06,70.75).

According to the effect of EW on hatchability indicators (p < 0.001), the highest hatching percentage (72.1%) was observed for eggs weighing between 12.0 and 12.9 gm; this was significantly higher (p < 0.05) than the hatching percentages observed for eggs weighing 11 gm (−29.2%) and >13 gm (−41.3%) (Table 2). In terms of dead embryo rate, eggs weighing between 11.0 and 11.9 gm had the highest (p < 0.05) rate: 22.2% (Table 2). However, eggs weighing between 12.0 and 12.9 gm had the lowest embryonic mortality rate (p < 0.05), (Table 2). The fertility rate of eggs grouped according to their weight was significantly higher (p < 0.05) for eggs weighing between 11.0–11.9 gm (88.9%) and 12.0–19.9 gm (88.9% and 83.3%, respectively). Fertility rates were lowest (p < 0.05) for eggs weighing more than 13 gm: 69.2% (Table 2). The regression estimators exhibited quadratic behavior when considering the distribution of EWs on the hatching rate (Fig. 1). The orthogonal polynomial analysis revealed that eggs weighing an average of 12.06 gm hatch at a greater rate: 70.7% (p < 0.05) (Fig. 1). 

**Table 2.** LS means for hatchability rate (%) according to EW.

Hatchability rate	EW (gm)			
	<11	11.0–11.9	12.0–12.9	>13
*N*	301	307	322	293
Hatched	42.9^a^	63.9^b^	72.1^c^	30.8^d^
Not hatched	14.3^a^	2.8^b^	8.3^c^	7.7^c^
Dead embryo	17.9^a^	22.2^b^	2.8^c^	15.4^a^
Fertile	25.0^a^	11.1b	16.7c	30.8^d^
Contaminated	0.0^a^	0.0a	0.0a	15.4^b^
SEM	3.3	3.3	3.2	5.9

^a,b,c ^Different literals indicate statistical difference (*p* < 0.05) within the row.

When the effect of the egg SI on hatchability indicators was evaluated (*p* < 0.001), it was discovered that eggs with a SI greater than 78% performed better in terms of hatching rate (21.9% higher hatching) and embryonic mortality (14.7% lower mortality), when compared to eggs with a SI less than 78% (*p* < 0.05) (Table 3). According to the SI, there was no difference between minced and unhatched eggs, nor was there a difference in fertilization rate (*p* > 0.05) (Table 3).

**Table 3.** LS means for hatchability rate according to the egg-shaped index.

Hatchability rate	Shape index (%)	
	<78	>78
*N*	300	300
Hatched	47.3^a^	67.6^b^
Not hatched	13.4^a^	13.6^a^
Dead embryo	20.2^a^	5.5b
Fertile	15.8^a^	13.3^a^
Contaminated	3.2	0.0
SEM	1.97	1.97

^a,b^ Different literals indicate statistical difference (*p* < 0.05) within the row.

## Discussion

The present study discovered that the color and shine of the eggshell and the weight and form index of the Japanese quail egg affected the hatchability indicators. The statistics accessible in the literature, on the other hand, are inconsistent in this regard. Zhao et al. [9] researched the effect of the color and area of eggshell spots on hatchability, establishing no association between the color of the shell and the size of the spots. In comparison, Galindez et al. [14] discovered a greater hatching percentage (48%) in eggs containing HP compared to eggs containing MP, which hatched at a rate of 42%. The variations between what these studies report could be attributable to a variety of factors, the primary one being the age of the birds. According to Lewko et al. [23], flock age has a considerable effect on eggshell coloration. In this inquiry, the eggs were obtained from birds of approximately identical age (a difference of 4 weeks), allowing for the omission of this component during the study.

Concerning the superiority of eggs with more pigmented patches in hatching, this has been related to the fact that these eggs remain in the female reproductive system for longer. This increases its resistance to infections that could disrupt embryonic development by increasing the thickness of the shell and the amount of cutin covering the pores [24]. Additionally, it has been proven that biliverdin, which results in blue-green patches on the eggshell, has antioxidant characteristics [25], while protoporphyrin boosts shell fracture resistance through increased myelization [26].

In terms of hatchability, the results obtained (75.70%) in eggs with a shining shell are comparable to those obtained by Zhao et al. [9]. These authors found incubation advantages for eggs with an SS, demonstrating a 79% hatchability and fertility. Galindez et al. [14] determined hatching disparities between viable eggs based on the sheen of the shell: 43.9% for eggs with an SS and 36.7% for eggs with an OS.

A protein may explain the increased hatchability of eggs with an SS- and lipid-rich cuticle covering the egg because the cuticle’s function is to regulate gaseous and moisture exchange with the environment. Thus, they contribute to the egg albumin’s quality and, as a result, to the egg’s acid-base balance, which may aid embryonic survival and subsequent hatching [9,27]. Additionally, while calcium carbonate is the primary component of the shell (96%), the remaining components include magnesium, phosphorus, copper, zinc, iron, and a variety of trace elements (lithium, strontium, among others) [24]. All of the correlations between shell color and hatchability are most likely attributable to the characteristics of the shell pigments, as well as the bioavailability of the constructive elements, which are color-dependent and affect the hatchability of the eggs [28]. However, this occurrence warrants further examination.

Along with the effect of the egg’s color on hatchability, there is evidence of a correlation between the pigmentation of the eggshell and its weight [29], which would account for the observed results (Table 2). Lembcke et al. [30] discovered a positive connection between EW and hatchability of 0.94% and hatchability of 75.5%, 86.2%, and 75.0% in quail eggs aged 5, 10, and 15 months, respectively. Because the eggs for this experiment were collected and hatched by 9-month-old quail, the hatchability results (72.1%) are lower than those reported by the authors (86.2%). Dere et al. [31] argue that a criterion for selecting fertile eggs is that they weigh at least 12 gm, a requirement that corresponds to the current study’s maximum hatching rate (Table 2).

Kartikayudha et al. [32] establish an average hatching EW of 11–13 gm, which occurs after the quail reaches the age of 2.5 months. The maximum hatching rate was reported in the present experiment between the recognized limits of 11–13 gm in eggs weighing 12.0–12.9 gm (Table 2). According to the computed regression equation (Fig. 1), the eggs weighing 12.06 gm had the highest hatching percentage (70.76%). A proposed explanation for the influence of EW on hatchability is that eggs of medium weight have a high capacity for moisture and gas exchange with the environment, owing to a more significant number of pores per unit of the shell surface. Ozbey and Ozcelik [33] demonstrate that a greater capability for gas and moisture exchange with the medium is directly associated with a higher quality of albumin, which assures the embryo’s survival.

Besides EW, morphological traits must be considered when selecting eggs for hatching, as they directly affect hatchability [31]. According to Birkhead et al. [34], the egg SI is a shape-determining variable, a ratio of the egg’s width to its length. According to this source’s standards and the findings of this study, the egg SI was round: 67.62%; yet, the same source states that the best eggs for hatching had an oval shape and an index of between 72% and 76%.

Kostaman and Sopiyana [35] assert that the egg’s SI affects its hatchability. Around 20% of the variance in the sorts of eggs that hatch best and worst is seen in those with the best form index. The normal form has the maximum hatchability, with values between 80% ± 8% of the form SI; this is 13% greater than reported in the current investigation. 

## Conclusion

Eggs with a medium to highly pigmented shell, eggs with a SS, eggs weighing between 12.0 and 12.9 gm, and eggs with a SI of more than 78% all contribute to increased hatchability efficiency.

## List of abbreviations 

EW: egg weight; HP: high pigmentation; LP: low pigmentation; MP: medium pigmentation; OS: opaque shell; SI: shape index; SS: shiny shell.
